# Bioactive Compounds of *Ilex paraguariensis*: A Critical Update on Extraction, Gastrointestinal Stability, and Technological Applications

**DOI:** 10.1111/1750-3841.70679

**Published:** 2025-11-07

**Authors:** Sandra Maria Schenatto Palavicini, Bruna Maria Saorin Puton, Rosangela Assis Jacques, Eunice Valduga, Geciane Toniazzo Backes, Natalia Paroul, Clarice Steffens, Rogério Luis Cansian

**Affiliations:** ^1^ Department of Food Engineering URI Erechim Rio Grande do Sul Brazil; ^2^ Chemistry Institute Federal University of Rio Grande do Sul (UFRGS) Porto Alegre Rio Grande do Sul Brazil

**Keywords:** bioactive compounds, extraction, functional foods, *Ilex paraguariensis*, simulated digestion, technological innovation

## Abstract

*Ilex paraguariensis* A. St. Hil., commonly known as yerba mate, is a native South American species widely used in traditional beverages such as chimarrão, terere, and mate tea. In recent years, this plant has attracted growing scientific and industrial interest due to its richness in bioactive compounds such as phenolic acids, flavonoids, methylxanthines, and saponins, which are associated with antioxidant, anti‐inflammatory, hypolipidemic, and neurostimulant properties. This review article provides a comprehensive and critical analysis of the botanical, genetic, and agronomic aspects of *I. paraguariensis*, also discussing how industrial processing, conventional and advanced extraction methods, and consumption forms influence its phytochemical and functional composition. Particular emphasis is given to studies on simulated gastrointestinal digestion, which reveal the bioaccessibility and stability of key compounds such as chlorogenic acid and caffeine. The review also explores the wide range of technological applications of yerba mate, spanning functional foods, cosmetics, pharmaceuticals, biodegradable packaging, and international patents. Current data highlight the potential of yerba mate as a functional and strategic ingredient for the development of safe and sustainable products, while also identifying relevant scientific gaps, such as the lack of comparative studies between cultivars and standardized extraction methods that need to be addressed to support health claims. By integrating multidisciplinary knowledge, this work provides solid foundations for the scientific and technological valorization of *I. paraguariensis*.

## 1 Introduction

*Ilex paraguariensis* A. St. Hil. (Aquifoliaceae), commonly known as yerba mate, is a plant native to the subtropical regions of South America, traditionally cultivated and consumed in Brazil, Argentina, Paraguay, and Uruguay. Used for centuries by Indigenous peoples such as the Guarani, its consumption remains deeply rooted in the culture of these countries, particularly in the forms of chimarrão, terere, and mate tea (Bracesco 2019). Today, yerba mate has transcended cultural and geographic boundaries, reaching international markets such as Germany, Japan, the United States, and France, driven by the appeal of its functional properties and nutraceutical potential (de Vasconcellos et al. 2022).

The growing global popularity of *I. paraguariensis* is largely attributed to its complex chemical composition. The leaves contain a wide range of secondary metabolites with biological activity, including phenolic acids, flavonoids, saponins, vitamins, minerals, and methylxanthines such as caffeine, theobromine, and theophylline (Gómez‐Juaristi et al. 2018; Budin et al. 2025). These compounds contribute to the plant's antioxidant, anti‐inflammatory, stimulant, and hypolipidemic properties, with studies suggesting protective effects against cardiovascular, neurodegenerative, and metabolic diseases (Bracesco 2019; Cardozo et al. 2021; Santos et al. 2022).

Agronomic factors such as cultivation system (shade‐grown or full sun), genotype selection, and management practices directly influence the plant's phytochemical profile. Improved cultivars like Cambona‐4, for instance, are valued for their productivity, higher concentration of bioactive compounds compared to native populations, milder flavor, and suitability for sustainable agroforestry systems (Correa et al. 2017).

Industrial steps such as sapeco, drying, grinding, and maturation influence both the stability of bioactive compounds and their subsequent release, absorption, and bioaccessibility during consumption (Calvo et al. 2025; Steffens et al. 2023). Most studies focus on traditional infusions such as chimarrão, terere, and mate tea, but innovative forms of preparation, such as yerba mate capsules compatible with espresso machines, have gained attention for their convenience and potential to enhance the extraction of bioactive compounds under standardized conditions. However, despite the growing body of evidence on the biological effects of yerba mate, important gaps remain concerning its digestibility and the actual absorption of functional compounds in the human body. In vitro gastrointestinal simulation studies have shown that compounds such as chlorogenic acids may remain stable during digestion, retaining their antioxidant activity even after enzymatic action (Pagliosa et al. 2021; Correa et al. 2017). Moreover, strategies such as microencapsulation in maltodextrin or calcium alginate have proven effective in protecting these compounds during gastrointestinal transit (Correa et al. 2017), broadening the possibilities for the use of yerba mate as a functional ingredient.

This review article aims to address this gap by compiling and critically analyzing the scientific literature on the chemical, industrial, and biological aspects of *I. paraguariensis*, with an emphasis on its bioactive compounds, extraction methods, and in vitro digestibility. Furthermore, this work proposes a novel discussion on the efficiency of new preparation formats in enhancing the release and absorption of functional compounds, with the goal of contributing to the development of innovative, safe, and technologically optimized products based on yerba mate.

## 2 Botanical, Genetic, and Agronomic Aspects of *Ilex paraguariensis*

*I. paraguariensis* A. St. Hil. is a perennial tree species from the Aquifoliaceae family, endemic to the subtropical regions of South America. Its natural distribution covers southern Brazil, northeastern Argentina, and Paraguay, predominantly in Mixed Ombrophilous Forests and Seasonal Deciduous Forests. In Brazil, it occurs spontaneously in the states of Paraná, Santa Catarina, Rio Grande do Sul, and to a lesser extent in Mato Grosso do Sul, playing an important role in both regional culture and the local economy (Cardozo et al. 2021; Valduga et al. 2023).

Under natural conditions, the plant can reach up to 20 m in height and features leathery, alternate, serrated leaves with a deep green color, which concentrate the main bioactive compounds of commercial interest. Its fruit is a globular red drupe containing a single seed, contributing to the natural regeneration of the species in forest environments (Vieira et al. 2008).

Historically used by Indigenous peoples, yerba mate is now cultivated under different production systems, which influence its chemical, sensory, and ecological characteristics. Full‐sun cultivation, widely adopted for its high productivity, promotes the accumulation of compounds such as polyphenols and xanthines and accelerates plant growth. However, it requires more intensive phytosanitary control and management practices such as irrigation, fertilization, and pruning to maintain leaf quality. In contrast, shaded cultivation, typical of forested areas or agroforestry systems, tends to preserve genetic diversity and yield more complex sensory profiles. It is particularly valued by consumers seeking a milder flavor and more sustainable production (Florestas 2025; Vieira et al. 2008).

The species' genetic diversity has been harnessed by breeding programs aimed at developing cultivars with higher productivity, adaptability, and concentration of functional compounds. Notable commercial cultivars include Cambona‐4, BRS 408, BRS 409, BRS BLD Yari, BRS BLD Aupaba, and SCSBRS Caa Rari. Cambona‐4, in particular, is a biclonal progeny known for its high yield, stress resistance, and smooth flavor, making it well‐suited for agroforestry systems and formulations requiring reduced bitterness (Correa et al. 2017).

The use of clonal seedlings derived from selected parent plants, combined with modern cultivation techniques, has contributed to the advancement of the yerba mate production chain by promoting product uniformity and increasing the concentration of bioactive compounds. Nevertheless, there is still a lack of commercial cultivars with guaranteed levels of functional compounds, which poses a challenge for the standardization and nutritional valorization of raw material in the market (Valduga et al. 2023).

The choice of cultivation system and cultivar directly influences the phytochemical content of the leaves and the stability of compounds throughout processing. Therefore, botanical and agronomic knowledge of *I. paraguariensis* forms the foundation for more efficient and sustainable cultivation strategies, aiming at the production of raw materials with greater functional value. This knowledge is essential for the development of new products and technological applications.

## 3 Processing, Industrialization, and Quality of *Ilex paraguariensis*

The processing of *I. paraguariensis* involves a complex technological chain that is essential for ensuring the sensory, chemical, and microbiological quality of the final product. Industrial steps directly influence the content of bioactive compounds such as flavonoids, methylxanthines, and phenolic acids, as well as the characteristic taste, color, and aroma of yerba mate‐derived beverages (Steffens et al. 2023).

**FIGURE 1:**
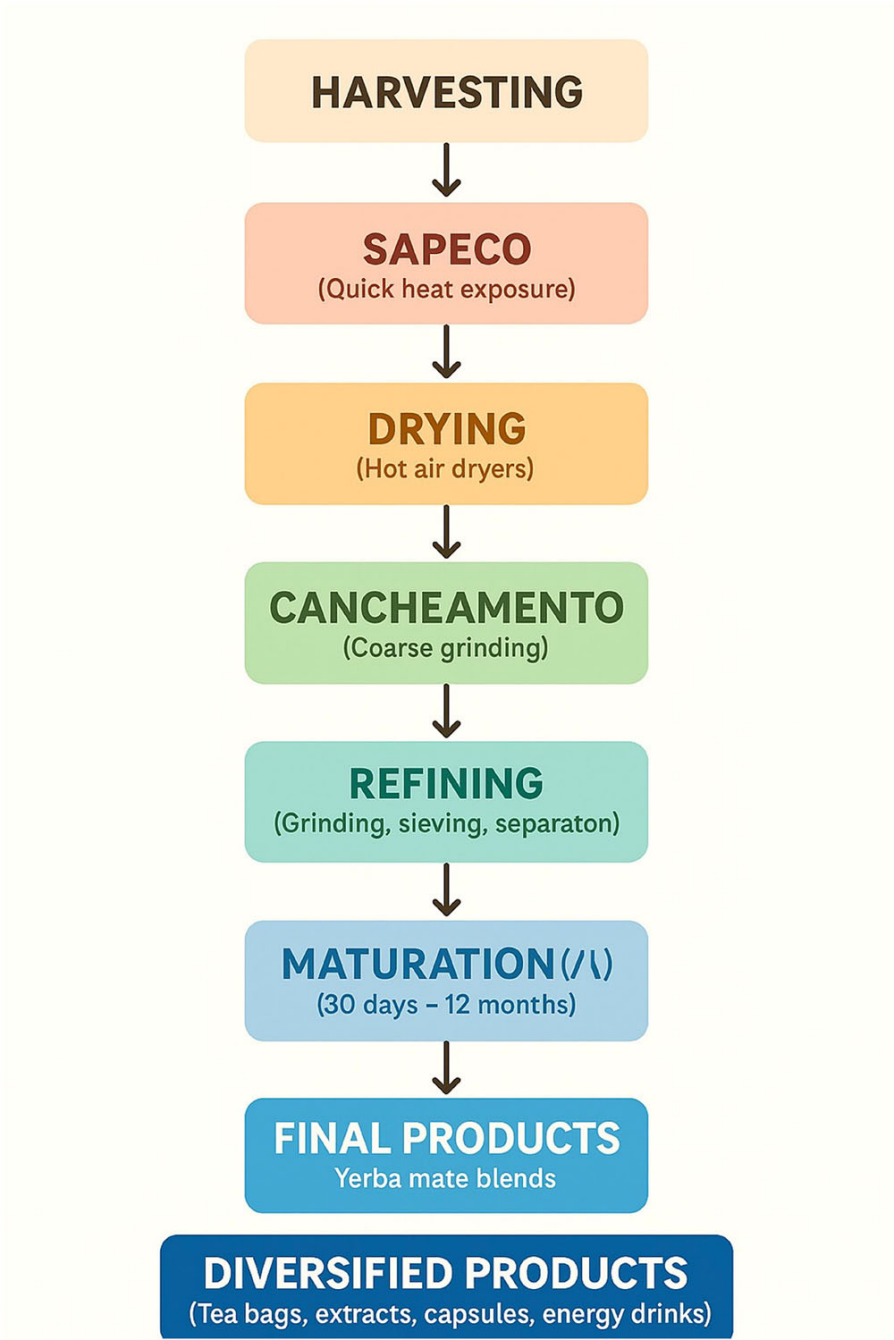
Flowchart of the industrial processing chain of *Ilex paraguariensis* and its influence on product quality.

After harvesting, the leaves and fine twigs undergo *sapeco*, a brief heat exposure step that inactivates oxidative enzymes such as peroxidases and polyphenol oxidases. A schematic flowchart summarizing the industrial processing chain of I. paraguariensis is presented in Figure 1. This prevents browning and the degradation of phenolic compounds, thereby preserving both sensory attributes and bioactive content (Calvo et al. 2025). Next comes drying, which is typically carried out using hot air in rotary or conveyor‐type dryers and has a direct impact on the product's phytochemical profile. Inadequate temperature or duration during this stage can reduce the functional quality of the plant and favor the formation of contaminants such as polycyclic aromatic hydrocarbons (Calvo et al. 2025; Cardozo et al. 2021).

The following step is *cancheamento*, in which the material is coarsely ground. This is followed by refining, which includes *soque* (grinding using mechanical pistons) to achieve the desired particle size, and the separation of leaves and petioles using sieves, blowers, and filters to ensure product uniformity. According to Brazil's ANVISA Resolution RDC No. 302/2002 (BRASIL 2002), the final composition of commercial yerba mate typically contains about 70% leaves and 30% fine stems.

Maturation, also referred to as aging or resting, is an optional yet important stage that contributes to the development of yerba mate's sensory profile. This process may last from 30 to 60 days or up to 12 months, depending on market preferences. In Brazil, consumers tend to prefer younger mate, whereas in Argentina and Uruguay, aged mate is favored for its enhanced flavor and reduced bitterness. Maturation does not significantly alter the levels of bioactive compounds but promotes chemical transformations that improve aroma and reduce undesirable volatile compounds (Isolabella et al. 2010; Montiel 2012).

Processing also affects the balance between the plant's main functional compounds. Studies show that *sapeco*‐treated yerba mate contains approximately 1.3 times higher levels of polyphenols and flavonoids compared to air‐dried mate, while air‐dried mate retains about twice the concentration of methylxanthines. These findings demonstrate how different processing methods can significantly shape the functional profile of the final product (Cardozo et al. 2021; Isolabella et al. 2010).

Technological advancements have enabled the diversification of yerba mate‐derived products, including tea bags, instant powders, concentrated extracts, capsules, and energy drinks. These products are made from raw materials subjected to thermal treatment, aqueous or hydroalcoholic extraction, and spray drying, aiming to concentrate the bioactive compounds.

Therefore, comprehensive technical control over the industrial processing stages of yerba mate, including precise thermal control, the selection of appropriate drying methods, and the application of efficient extraction techniques, is essential for producing high‐quality products with desirable functional and sensory characteristics. Standardized processing, thermal control, and innovations in drying and extraction technologies are key factors in enhancing the commercial value of *I. paraguariensis* and in developing safe, high‐added‐value products for the food, cosmetic, and pharmaceutical industries.

## 4 Yerba Mate Products and Applications: Tradition and Innovation

Yerba mate exhibits wide applicability across the food, cosmetic, pharmaceutical, and chemical industries, establishing itself as a functional ingredient with high bioactive value. Traditionally consumed in beverages such as chimarrão, terere, and mate tea, *I. paraguariensis* is increasingly being incorporated into new formats and industrial uses, expanding its presence in both domestic and international markets (Cardozo et al. 2021; Ohtaki et al. 2023).

Traditional consumption methods, such as chimarrão (hot infusion) and terere (cold infusion), are especially appreciated in Mercosur countries. Mate tea, obtained from toasted leaves, is widely consumed in Brazil, both as a hot infusion and as an instant iced beverage (Mesquita et al. 2021). These drinks are rich in bioactive compounds such as polyphenols, flavonoids, and methylxanthines, which are associated with antioxidant, anti‐inflammatory, and central nervous system‐stimulating effects (da Silveira et al. 2021; Santos et al. 2022).

In contrast to these traditional formats, recent innovations have introduced modern methods of consumption designed to meet contemporary demands for convenience, portability, and product diversification. With the growing demand for functional and convenient foods, yerba mate has been incorporated into innovative products such as espresso‐machine‐compatible capsules (e.g., Chimarrão Expresso), energy drinks, soft drinks, beers, liqueurs, syrups, snacks, sweets, fiber‐enriched candies, and even functional cheeses (Cardozo et al. 2021; Ohtaki et al. 2023). These newer forms differ significantly in preparation and usage from traditional infusions, often relying on industrial processing, standardized dosages, and extended shelf life. These innovations aim to maintain, or even enhance, the delivery of bioactive compounds to consumers, combining flavor, convenience, and health benefits.

The development of pressurized yerba mate capsules represents a major advancement by allowing more standardized and efficient extractions, with high release rates of compounds such as caffeine, theobromine, and chlorogenic acids, even after multiple infusions (Panzl et al. 2022). Compared to the manual and time‐consuming preparation of traditional infusions, capsule‐based systems offer rapid preparation and reproducible dosing, making them more accessible to new consumer groups. Specific grinding techniques and temperature control during industrial processing help preserve sensitive compounds and facilitate their release during infusion, suggesting promising applications in the functional beverage sector.

Beyond beverages, yerba mate has been used as a natural antioxidant in dipping solutions to control enzymatic browning in fresh fruits and as an active component in biodegradable films with preservative properties, demonstrating its potential as a substitute for synthetic additives (Croge et al. 2021).

In the cosmetics industry, yerba mate extracts are incorporated into shampoos, soaps, moisturizers, and antiaging products due to their antioxidant, astringent, and photoprotective actions. In the chemical industry, these extracts are used in the manufacture of resins, paints, textile dyes, and personal care products (Cardozo et al. 2021). Another promising avenue involves the reuse of by‐products from the production chain. Shells, processing residues, and leftover leaves can be used as organic fertilizers or animal feed, adding value to waste and promoting agro‐industrial sustainability (Croge et al. 2021).

Table 1 summarizes the main applications of yerba mate, highlighting the comprehensive use of its parts (leaves, twigs, fruits, seeds, and residues) across various production chains.

**TABLE 1:** Applications of *Ilex paraguariensis* (yerba mate) in different sectors, consumption formats, and patented products.

Plant part/derivative	Application mode	Examples and uses
**Leaves and twigs**	Traditional uses (beverages)	Chimarrão, terere, mate tea (toasted leaves), herbal blends, flavored teas.
	Nontraditional uses (beverages)	Freeze‐dried extract, green leaf tea, tea capsules, milk‐infused mate, energy drinks, beers, soft drinks, liqueurs.
	Functional foods	Candies, jams, breaded products, functional cheeses, ice creams, snacks, fortified cookies.
	Cosmetics	Shampoos, soaps, moisturizing and antiaging creams, body lotions, natural exfoliants.
	Pharmaceutical products	Raw material for herbal medicines and antioxidant supplements.
	Natural antioxidant additive	Aqueous dipping solutions to prevent browning in fresh fruits; biodegradable edible films for fruit packaging.
	Textile industry	Natural dye for silk, wool, linen, and cotton fabrics.
**Fruits**	Cosmetics	Essential oil with antioxidant potential; used in antiaging and moisturizing creams.
	Biological control	Extracts from green fruits with molluscicidal activity.
**Seeds**	Propagation and environmental restoration	Seedlings for afforestation, landscaping, and ecological restoration of degraded areas.
**Wood**	Timber industry	Production of veneer sheets and other wood‐based products.
**Processing residues**	Agriculture	Organic fertilizer, agricultural substrates, alginate‐based compost for controlled nutrient release (*Patent BR102014021764A2*).
	Energy	Conversion into biochar or biomass for thermal energy.
	Livestock	Nutritional supplement for animal feed.
**Extracts and bioactive compounds**	Patented cosmetic formulations	*Patent WO2010118489A1*: yerba mate extracts used in antioxidant lotions and antiaging creams.
	Patented functional beverages	*Patent CN104605456A*: banana‐flavored yerba mate syrup for functional and energy drinks.
	Nutraceutical compositions	*Patent EP2609945B1*: yerba mate‐based supplement for appetite regulation, lipid metabolism, and glycemic control.
	Antioxidant food additive	*Patent US20140212467A1*: application of yerba mate phenolic compounds as natural preservatives in food products.

*Source*: Adapted from Croge et al. (2021), with updated data.

The versatility of yerba mate, combined with its functional composition and natural appeal, positions the species as a strategic resource for technological innovation in foods, cosmetics, packaging, and dietary supplements. This wide range of applications highlights the importance of studies that correlate extraction processes, processing methods, and the bioaccessibility of bioactive compounds, aiming to enhance health benefits while meeting modern consumer demands for more natural, sustainable, and functional products.

## 5 Addition of *Ilex paraguariensis* in Foods: Stability, Consumer Health Effects, and Sensory Aspects

The incorporation of *I. paraguariensis* into food products has emerged as a promising strategy to deliver bioactive compounds to consumers in a practical and appealing way. Its addition provides technological and functional advantages, but three factors are decisive for the success of such formulations: the stability of bioactive compounds during processing and storage, the effects on consumer health, and the impact on sensory quality and acceptance. The stability of phenolic acids, flavonoids, and saponins from yerba mate is directly influenced by the food matrix, processing conditions, and storage environment. Thermal treatments such as baking or pasteurization tend to degrade phenolic acids like chlorogenic acid, although methylxanthines such as caffeine and theobromine remain relatively stable (Budin et al. 2025; Fioroto et al. 2022). In bakery products, fortification with yerba mate generally preserves 50%–70% of the initial polyphenolic content after baking, while encapsulation techniques, such as spray drying with maltodextrin or ionic gelation with alginate can increase retention to levels above 80%, ensuring higher antioxidant activity in the final product (Budin et al. 2025; Santetti et al. 2022). These protective strategies not only enhance stability but also improve compound release during digestion, expanding their functional potential. Interactions with proteins and polysaccharides in dairy matrices, for example, may contribute to stabilization of phenolic compounds, although in some cases they can also limit immediate bioavailability (Croge et al. 2021; Najman et al. 2024).

In terms of consumer health, foods enriched with yerba mate have demonstrated improvements in antioxidant status and beneficial effects on lipid and glucose metabolism. Clinical and experimental studies indicate that the regular intake of yerba mate‐fortified products can lower LDL cholesterol, increase HDL cholesterol, and enhance insulin sensitivity, effects mainly attributed to chlorogenic acid and saponins (Bracesco 2019; Gómez‐Juaristi et al. 2018). Fortification of breads, cookies, and dairy products with yerba mate extracts has been shown to delay lipid oxidation, reduce oxidative stress markers, and support cardiometabolic health (Santetti et al. 2022). In addition, the presence of caffeine and theobromine contributes mild neurostimulant effects such as enhanced alertness and reduced fatigue, without the excessive excitability often reported for coffee (Meinhart et al. 2010). Experimental models also suggest potential roles in modulating gut microbiota and inhibiting digestive enzymes such as pancreatic lipase, which reinforces the application of yerba mate as a functional ingredient with broad physiological benefits (Fayad et al. 2020; Santos et al. 2022).

The sensory aspects of yerba mate addition remain a critical point for consumer acceptance. The characteristic bitterness, astringency, and herbal aroma tend to intensify with increasing concentrations, particularly in bakery and dairy products. Additions above 3% frequently result in darker coloration and bitter aftertaste, which reduce acceptance in consumer panels. However, lower concentrations, usually around 1%–2%, are well tolerated and in some cases positively perceived, especially when combined with sweet or fruit‐based formulations (Croge et al. 2021; Santetti et al. 2022). In dairy matrices such as yogurts and cheeses, encapsulation technologies have proven valuable not only for preserving compounds but also for masking undesirable flavors, thereby improving palatability (Budin et al. 2025). In beverages, yerba mate is often well accepted due to its refreshing character and stimulating properties, provided that bitterness is balanced with sweeteners or complementary flavors. Consumer studies also reveal that health‐oriented marketing strategies, highlighting yerba mate as a natural source of antioxidants and energy, enhance acceptance even in markets where traditional consumption is not established (Ohtaki et al. 2023).

Altogether, the incorporation of *I. paraguariensis* into foods offers a valuable opportunity to combine technological innovation, health‐promoting effects, and sensory appeal. Nevertheless, the development of successful formulations requires careful optimization of concentration levels, processing conditions, and masking techniques, along with robust evidence of bioactive stability and efficacy. Advances in encapsulation, formulation design, and consumer‐driven product development are expected to strengthen the role of yerba mate as a strategic ingredient in the global functional food sector.

## 6 Phytochemical Profile and Functional Properties

Yerba mate stands out for its rich phytochemical profile and is considered a relevant source of bioactive compounds with well‐recognized functional properties. The leaves of *I. paraguariensis* primarily contain polyphenols, flavonoids, phenolic acids, methylxanthines (such as caffeine, theobromine, and theophylline), and triterpene saponins, in addition to vitamins, minerals, and pigments (Budin et al. 2025; Gómez‐Juaristi et al. 2018).

Among phenolic compounds, chlorogenic acids, especially the 3‐, 4‐, and 5‐caffeoylquinic acid isomers, are the most abundant. These contribute significantly to antioxidant activity and are linked to anti‐inflammatory and hypolipidemic effects (Bracesco 2019; Pagliosa et al. 2021). The predominant flavonoids include rutin, quercetin, and their glycosylated derivatives, which act as free radical scavengers and enzyme modulators in inflammatory and metabolic pathways (da Silveira et al. 2021).

Methylxanthines, particularly caffeine, are found in concentrations ranging from 0.3% to 1.8% of dry weight, depending on the cultivar, growing region, and processing method (Duarte et al. 2022; Gómez‐Juaristi et al. 2018). These compounds are responsible for the beverage's stimulant and thermogenic effects, as well as its characteristic bitterness.

For context, yerba mate typically contains about 78–196 mg of caffeine per 500 mL serving, depending on preparation, which is generally lower than a standard 240 mL cup of filtered coffee, which contains about 95–200 mg. In terms of chlorogenic acids, yerba mate infusions may contain between 20 and 40 mg per 100 mL, which is comparable to coffee, although coffee tends to have higher total chlorogenic acid content (70–350 mg per cup), depending on roast and brewing method. Such comparisons help to position yerba mate as a competitive alternative to coffee, offering similar stimulant and antioxidant properties with a distinct phytochemical profile (USDA 2018).

Triterpene saponins, present in concentrations up to 4%, exhibit anti‐inflammatory, antioxidant, and hepatoprotective activities and contribute to serum cholesterol modulation (Cardozo Junior and Morand 2016). They also play a role in the characteristic foam formation seen in chimarrão.

The content and balance of these bioactive compounds vary according to genetic factors (cultivar), edaphoclimatic conditions, maturation stage, cultivation methods (Isolabella et al. 2010; Steffens et al. 2023). Therefore, standardizing processing conditions and selecting appropriate extraction methods are essential to ensure the functional value of yerba mate‐based products.

A variety of extraction methods have been applied to optimize the recovery of bioactive compounds from yerba mate, ranging from traditional techniques like infusion and decoction to advanced approaches with precise control of time, temperature, and solvent use, such as ultrasound‐assisted extraction (UAE), eutectic solvent extraction, Soxhlet extraction, and pressure‐assisted extraction via espresso‐type capsules (dos Santos Polidoro et al. 2023; Najman et al. 2024).

Techniques using moderate temperatures (60–80°C) in combination with hydroalcoholic or eutectic solvents have shown high total phenolic yields and strong antioxidant activity. On the other hand, mild methods such as cold infusions with ultrasonication allow selective extraction of phenolic acids, though with lower total compound yields (Najman et al. 2024).

UAE has demonstrated high efficiency in releasing caffeoylquinic acids, flavonoids, and caffeine in short times, making it a promising alternative due to reduced solvent usage and improved preservation of sensitive compounds (dos Santos Polidoro et al. 2023). Although Soxhlet extraction is more aggressive and less sustainable, it is often used as a reference method due to its ability to maximize compound yields (Feihrmann et al. 2022; Fioroto et al. 2022).

The type of beverage (chimarrão, terere, or mate tea), preparation conditions (temperature and infusion time), and particle size directly influence the levels of extracted compounds. Comparative studies reveal that chimarrão contains the highest levels of chlorogenic acids, followed by terere and roasted mate (Fioroto et al. 2022), while infusion temperature can significantly affect the extraction of flavonoids and methylxanthines (Silveira et al. 2021).

New approaches, such as the use of extraction at high pressure are gaining attention for providing rapid, standardized extractions with efficiency comparable to conventional methods. Studies show that pressure‐based extraction can achieve up to 90% release of functional compounds after three successive infusions, making it a promising technique for both laboratory and commercial applications (Panzl et al. 2022). Table 2 compiles comparative data on yerba mate extraction methods, highlighting the influence of parameters such as temperature, time, and solvent type on the efficiency of functional compound recovery, with potential applications in the food, cosmetic, and nutraceutical sectors.

**TABLE 2:** Different extraction methods of bioactive compounds from yerba mate, with time and temperature conditions and bioactive compound yields.

Extraction method	Time and temperature	Bioactive compounds extracted	Refs.
			
Eutectic solvent extraction	60°C for 60 min	Total phenolics: 1080 mg GAE/g; flavonoids: 0.20 mg QE/g; antioxidants: 1080 mg GAE/g; saponins: 0.20; caffeine: 0.20 mg/g	dos Santos Polidoro et al. (2023)
Ultrasound‐assisted extraction	80°C for 11.6 min	TPC: 31.6–56.2 mg GAE/g; TFC: 6.47–10.49 mg RE/g; 3‐CQA: 28.9–37.81 mg/g; FQA: 0.11–0.82 mg/g; rutin: 6.47–10.49 mg/g; caffeine: 7.9–16.05 mg/g	Duarte et al. (2022)
Aqueous extraction	70°C for 30 min	Total phenolics: 73.9 mg GAE/g; caffeine: 8.8–20.4 mg/g; antioxidant activity (ABTS): 886 µmol trolox/g; DPPH: 588.1 µmol trolox/g	Fioroto et al. (2022)
Aqueous extracts	80°C/20°C/90°C for 5 min	Varied chlorogenic acids and flavonoids; total polyphenols: chimarrão: 21.90 mg/kg; terere: 17.51 mg/kg; mate tea: 16.11 mg/kg	Feihrmann et al. (2022)
Soxhlet with ethanol	50°C for 12 h	Total phenolics: 644.17 ± 0.08 mg GAE/g; antioxidant (DPPH): 111.25 ± 0.04 µmol trolox/g	Santos et al. (2022)
Soxhlet with water	60°C for 6 h	Total phenolics: 236.28 ± 11.83 mg GAE/g; flavonoids: 44.07 ± 5.56 mg QE/g	Vanin dos Santos Lima et al. (2022)
Hot infusion	Boiling water for 20 min	Chlorogenic acid: 42%; gallocatechin: 21%; gallic acid: 11%; caffeine: 8%	de Vasconcellos et al. (2022)
Hot infusion	70°C for 5 min	Total phenolics: 1200 mg GAE/g; flavonoids: 200 mg QE/g; antioxidants: 1500 µmol trolox/g; caffeine: 1800 mg/g; theobromine: 100 mg/g	de Brito et al. (2021)
Ethanol + water extraction	25°C for 24 h	Phenolics: 15.9 mg/L; flavonoids: 27 mg/L; antioxidants: 97 mg/L	Cardozo et al. (2021)
Decoction	100°C for 15 min	Various chlorogenic acids (26.8%–28.8%, 21.1%–22.4%, etc.); rutin: 7.1%–7.8%	Pagliosa et al. (2021)
Cold water infusion	25°C for 2.5–10 min	3‐CQA: 1.36 mg/g; 4‐CQA: 1.09 mg/g; 5‐CQA: 0.78 mg/g; 3,5‐di‐CQA: 0.56 mg/g; 4,5‐di‐CQA: 0.45 mg/g	de Brito et al. (2021)
Hot water infusion	70°C and 90°C, 5–30 min	Phenolics: 59.25–62.25 mg/100 g; flavonoids: 3.06–757.00 mg/100 g; anthocyanins: 17.52–43.79 mg/100 g; rutin: 1.86–6.10 mg/g	Croge et al. (2021)
Trifluoroacetic acid + methanol	45°C for 6 min	Phenolics: 104.54 g GAE/g; flavonoids: 23.11 g QE/g; antioxidants (DPPH): IC_50_ = 7.91 µg/mL; FRAP: 4922.67 µmol Fe(II)/g; caffeine: 25 mg/g	Tonet et al. (2019)
Hot water + steam pressure and spray‐drying	96°C for 5–10 min	Total phenolics: 1.39–2.81 mg GAE/mL; flavonoids: 73.33–112.64 µg RE/mL; caffeic acid: 1.19–2.49 µg/mL	Cazal (2019)
Hot infusion	60°C for 10 min	Total phenolics: 65.5 ± 2.6 mg/100 mL	Zenaro et al. (2014)
Water infusion	60/75/90°C for 5/7.5/10 min	Total phenolics: 349.92–428.31 mg GAE/L; flavonoids: 268.06–421.75 mg CTE/L; antioxidants (DPPH): 52.38%–80.65% inhibition	Bassani et al. (2014)

The choice of extraction method should consider not only the total content of compounds, but also the desired phytochemical profile, the thermal stability of the compounds, the type of plant matrix, and the intended application (nutraceutical, food, cosmetic, or pharmaceutical). Therefore, optimizing extraction conditions is a strategic step to maximize the functional value of yerba mate across different applications.

## 7 Health Benefits Associated With *Ilex paraguariensis*

Yerba mate has been extensively studied for its functional and therapeutic potential, which is attributed to the synergistic action of secondary metabolites such as phenolic acids (particularly chlorogenic acids), flavonoids, methylxanthines (caffeine and theobromine), and saponins. These compounds confer to *I. paraguariensis* antioxidant, anti‐inflammatory, hypolipidemic, neurostimulant, antimicrobial, and gastroprotective properties (Bracesco 2019; Cardozo Junior and Morand 2016; Fioroto et al. 2022)

### 7.1 Antioxidant and Anti‐Inflammatory Activity

The ability of yerba mate to neutralize free radicals has been widely documented and is primarily attributed to the presence of chlorogenic acids and flavonoids such as rutin. These compounds enhance the activity of endogenous antioxidant enzymes and reduce markers of oxidative stress, exerting beneficial effects on cellular integrity, liver tissues, and the cardiovascular system (Colpo et al. 2016).

Its anti‐inflammatory activity is also well documented in the literature, with studies demonstrating the inhibition of inflammatory mediators and cytokines such as TNF‐α and IL‐6, as well as the modulation of intracellular pathways like NF‐κB (da Silveira et al. 2021; Fioroto et al. 2022). These effects contribute to the prevention of chronic diseases, including obesity, metabolic syndrome, and neurodegenerative disorders.

### 7.2 Effects on Lipid and Glucose Metabolism

Multiple studies indicate that regular consumption of yerba mate can improve lipid profiles by reducing LDL cholesterol and increasing HDL levels, in addition to attenuating hepatic steatosis and insulin resistance (Fioroto et al. 2022; Gómez‐Juaristi et al. 2018). These effects are mainly attributed to compounds such as chlorogenic acid, which inhibits key enzymes involved in lipid metabolism, such as pancreatic lipase, and modulates the expression of genes regulating energy homeostasis, including AMPK (Bracesco 2019). In human studies, the consumption of yerba mate infusions has been shown to reduce LDL cholesterol levels by up to 13% in individuals with hypercholesterolemia, particularly when used alongside conventional treatments (de Morais et al. 2009).

### 7.3 Neurostimulant Activity and Cognitive Effects

The methylxanthines found in yerba mate, such as caffeine and theobromine, are responsible for its stimulating effects on the central nervous system, enhancing alertness, improving cognitive performance, and reducing mental fatigue (Meinhart et al. 2010). Theobromine, in particular, has a longer‐lasting and milder action compared to caffeine, contributing to the popularity of chimarrão and terere as alternatives to coffee. Moreover, there is evidence suggesting that yerba mate may exert neuroprotective effects, possibly associated with the reduction of reactive oxygen species and the modulation of neurotransmitters and neurotrophic factors (Bracesco 2019).

### 7.4 Antimicrobial and Gastroprotective Effects

Yerba mate extracts exhibit significant antimicrobial activity against pathogenic microorganisms such as *Salmonella* spp., *E. coli*, *Staphylococcus aureus*, and *Candida albicans*, due to the presence of tannins, phenolic acids, and saponins (Fayad et al. 2020). This activity may help support intestinal microbiota balance and contribute to digestive health. In experimental models, gastroprotective effects have also been reported, including reduced gastric lesions induced by irritant agents and protection of the gastric mucosa. These effects are likely related to the plant's antioxidant capacity and its ability to modulate gastric pH and mucus secretion.

### 7.5 Potential in the Prevention of Chronic Diseases

**FIGURE 2:**
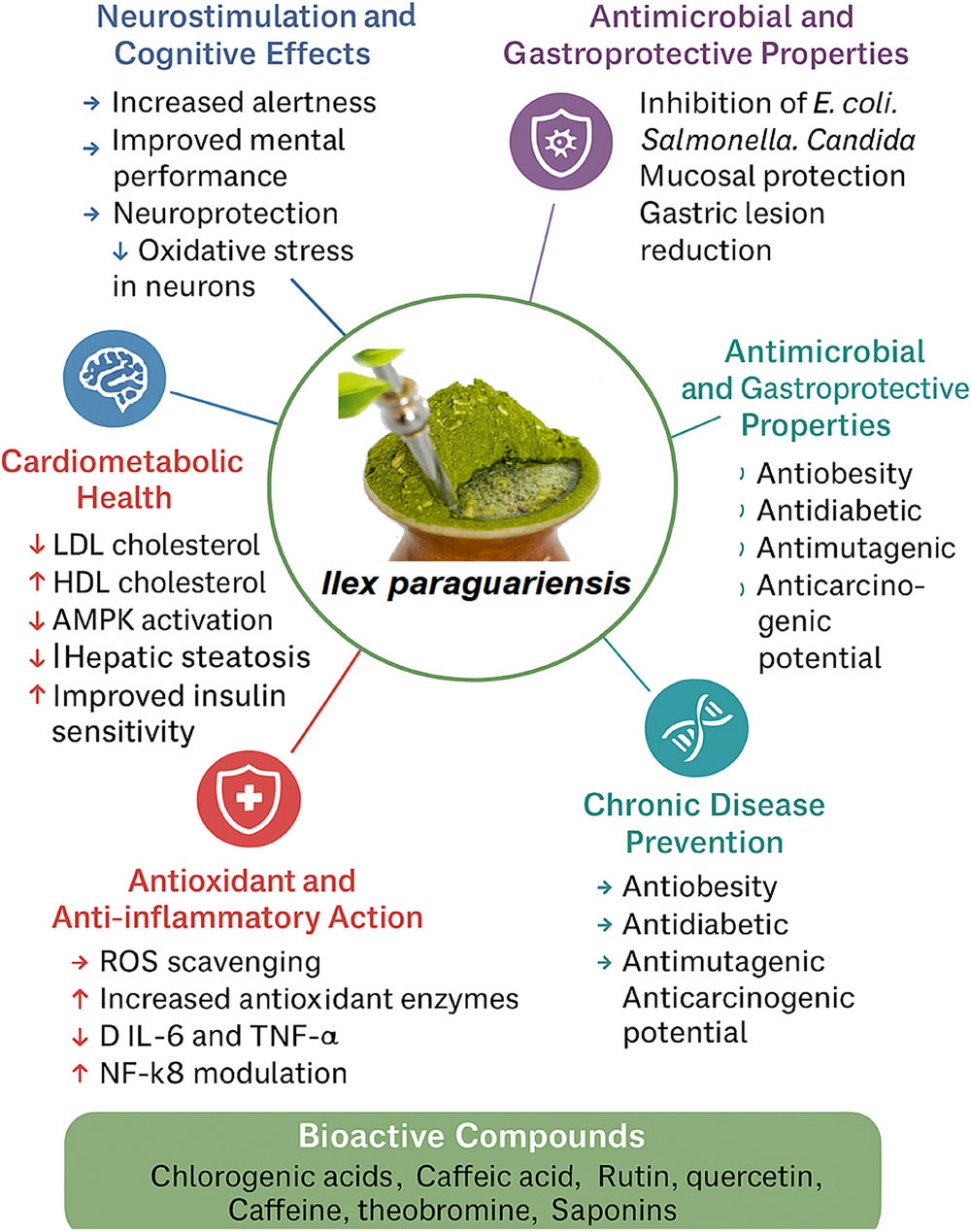
Health‐promoting effects of *Ilex paraguariensis* (yerba mate).

In addition to the aforementioned benefits, yerba mate bioactive compounds have been investigated for their antidiabetic, antiobesity, anticancer, and antimutagenic effects. The activation of metabolic pathways such as AMPK, inhibition of adipogenesis, and modulation of inflammatory cytokines suggest that regular consumption of the beverage may contribute to the prevention of metabolic and degenerative diseases (Cardozo Junior and Morand 2016; Gómez‐Juaristi et al. 2018). Figure 2 summarizes the main physiological effects of yerba mate on parameters related to metabolic health, inflammation, and oxidative stress, with emphasis on its role in cholesterol regulation, energy metabolism, and liver protection.

### 7.6 Typical Intake Levels and Safety Considerations

The amount of yerba mate typically consumed can vary greatly depending on cultural practices and product format. In traditional forms such as chimarrão or terere, intake can reach 1–2 L per day, which may correspond to approximately 100–300 mg of caffeine and 300–600 mg of total chlorogenic acids daily, depending on the strength of infusion and frequency of refilling (Bracesco 2019; Heck and Mejia 2007).

In contrast, newer industrialized forms such as capsules, energy drinks, and extracts often contain standardized doses of caffeine and other bioactive compounds. These formats may deliver concentrated levels in smaller volumes, making it easier to exceed traditional intake thresholds, particularly when consumed alongside other caffeine‐containing products (Bastos et al. 2005).

Currently, there is no globally established recommended daily intake for yerba mate. However, several human trials of beneficial effects have employed intake levels or extract doses corresponding to about 1 L of infusion per day or 3 g of extract per day, with generally good tolerability in healthy adults (e.g., 3 g/day in a 12‐week supplementation trial; 1 L/day infusion in an 8‐week intervention; Kim et al. 2015). Therefore, while yerba mate offers promising functional benefits, its consumption should be mindful of caffeine sensitivity, preparation method, and potential interactions with other stimulants or medications.

## 8 Simulated Gastrointestinal Behavior and Bioaccessibility of Bioactive Compounds

Although *I. paraguariensis* is widely recognized as a source of bioactive compounds, the health effects of these metabolites depend not only on their concentration in the plant or final product, but also on their bioaccessibility that is, the fraction effectively released, absorbed, and utilized by the body. In this context, in vitro simulated gastrointestinal digestion studies have gained prominence, as they allow for the evaluation of compound behavior during digestive transit and provide estimates of their stability, solubility, and absorption potential (Pagliosa et al. 2021).

Simulated gastrointestinal digestion involves exposing yerba mate extracts to physiological conditions that mimic the oral, gastric, and intestinal compartments, including pH changes, the addition of digestive enzymes (pepsin, pancreatin), bile salts, and specific retention times. This methodology helps identify which compounds remain intact, which degrade, and which become more bioaccessible throughout the digestive process (Minekus et al. 2014).

Recent studies show that chlorogenic acids, flavonoids, and methylxanthines present in yerba mate maintain good stability during gastric and intestinal digestion. For example, Pagliosa et al. (2021) demonstrated that antioxidant activity of the extracts remained high after intestinal simulation, suggesting functional preservation of key compounds even after enzyme exposure and pH variation.

Furthermore, preparation methods such as hot infusions, pressurized capsules, and microencapsulated extracts can influence the degree of release and protection of bioactives during digestion. Microencapsulation with maltodextrin or calcium alginate has proven effective in protecting flavonoids and phenolic acids, prolonging their stability and enabling controlled release throughout the gastrointestinal tract. The type of compound is also a relevant factor: while methylxanthines exhibit high solubility and rapid absorption, phenolic acids may undergo partial hydrolysis or bind to food macromolecules via hydrogen bonding, which limits their intestinal absorption. Still, the nonabsorbed fraction may exert beneficial effects in the colon by modulating the gut microbiota and exerting local antioxidant action (Bracesco 2019; Najman et al. 2024).

The use of in vitro digestion models is a valuable tool for the development of functional yerba mate‐based products, enabling adjustments in formulation, solvent selection, particle size, and encapsulation techniques to enhance the release and absorption of target compounds. Table 3 summarizes the main findings from studies evaluating the bioaccessibility, stability, and biological activity of *I. paraguariensis* bioactives after simulated gastrointestinal digestion, considering different extract types and food matrices.

**TABLE 3:** Bioactive compounds from *Ilex paraguariensis*: Bioaccessibility, stability, and activity after simulated gastrointestinal digestion.

Extract/matrix	Bioactive compounds	Stability/bioaccessibility	Postdigestion activity	Refs.
Yerba mate extract	Phenolic compounds	Chlorogenic acid: ∼33% Gallic acid: ∼12% 3,4‐Dihydroxybenzoic and *p*‐coumaric acids: ∼10%–20%	Proportional reduction after digestion	Bremer Boaventura et al. (2015)
Colonic phenolic acids derived from *Ilex*	Phenolic compounds; Total flavonoids	Caffeoylquinic acids: ∼94%–100% Caffeoylglucose: ∼94.61% 3‐CQA: ∼52.97% Lactones: ∼99% (gastric), ∼38%–40% (intestinal) Flavonols: ∼96%	High gastric stability; partial intestinal reduction	Baeza et al. (2017)
Yerba mate beverages	Phenolic compounds	Chlorogenic acid: ∼33% absorbed in the small intestine; ∼67% reaches colon for metabolism	Conversion into colonic metabolites with potential bioactivity	Correa et al. (2017)
Mate tea	Total phenols; Chlorogenic acids; Flavonoids; Caffeine; Theobromine	Total phenols: ∼64% Chlorogenic acids: ∼70% Flavonoids: ∼59% Caffeine: ∼80% Theobromine: ∼78%	High stability of caffeine and theobromine	Cazal (2019)
Extract adsorbed on *S. cerevisiae*	Total phenols; Caffeine	Total phenols: ∼55.5% Caffeine: ∼60% (+239% vs. crude extract)	Increased postbiosorption in *S. cerevisiae*	Ribeiro et al. (2019)
Infusion of mixed plant species including *Ilex*	Phenolic compounds (e.g., NDGA, DHC, DHMC)	∼21%–50%, depending on composition and specific compounds; HM2 blend showed highest retention	Antioxidants preserved after digestion; not specific to *Ilex*	Carabajal et al. (2020)
Yerba mate leaf extract	Total phenols	∼20%–50%, depending on treatment, for example, 47% (250YM), 50% (500YM)	Significant reduction after digestion	Feihrmann et al. (2022)
Aqueous extract of yerba mate	Total phenols; Caffeine	Phenols: ∼20%–50% Chlorogenic acid: ∼30%–40% Caffeine: ∼45%–50%	Caffeine more stable than phenols	Monteiro et al. (2022)
Hydroalcoholic extract Aqueous extract (infusion)	Total phenols; Chlorogenic acid; Caffeine	Chlorogenic acid: ∼30%–35% Total phenols: ∼25%–40% Caffeine: ∼50%–60%	Greater bioaccessibility of caffeine	Clemente (2024)
Infusion of roasted yerba mate in milk	Phenolic compounds (chlorogenic acid, caffeic acid, rutin, quercetin)	Chlorogenic acid showed higher bioaccessibility (∼50%–60%) after digestion	Bioaccessibility influenced by protein–polyphenol interactions and bioactive peptide formation	Kautzmann et al. (2024)

The analysis of studies presented in Table 3 reveals considerable variability in the bioaccessibility of *Ilex paraguariensis* bioactive compounds, influenced by multiple factors such as compound type, food matrix, and the method of preparation or encapsulation. In general, compounds like caffeine and theobromine exhibit higher stability throughout simulated gastrointestinal digestion, often maintaining bioaccessibility above 50%. In contrast, phenolic compounds, especially hydroxybenzoic and hydroxycinnamic acids, show greater degradation, with values frequently ranging between 20% and 40%, depending on the study and the experimental protocol. These differences in experimental protocols include variables such as the type of digestion model used (e.g., static vs. dynamic), the pH and enzyme concentrations applied, the duration of each digestion phase, and the inclusion of colonic fermentation stages. For instance, the use of milk as a matrix was shown to enhance the bioaccessibility of chlorogenic acid due to protein–polyphenol interactions (Kautzmann et al. 2024), while biosorption on *Saccharomyces cerevisiae* significantly increased phenol and caffeine retention compared to crude extracts (Ribeiro et al. 2019). Gastric versus intestinal phase separations also influenced results where caffeoylquinic acids showed near‐complete stability in the gastric phase but notable reduction in the intestinal phase (Baeza et al. 2017).

Some specific phenolics, such as chlorogenic acid, although more prone to degradation during digestion, demonstrate moderate retention, particularly when incorporated into complex matrices such as milk or when protected by technological strategies like microencapsulation or yeast‐based biosorption. These approaches appear to confer resistance to degradation, resulting in greater retention of bioactive compounds and partial maintenance of antioxidant activity. Conversely, studies involving simple infusions or aqueous extracts report greater losses of phenolic compounds during digestion, although they sometimes lead to the formation of colonic metabolites with potential bioactivity, as observed in fermentation models.

Some specific phenolic compounds, such as chlorogenic acid, are prone to degradation during digestion but can show moderate retention depending on the food matrix and processing method. For instance, when yerba mate is consumed with milk, higher bioaccessibility of chlorogenic acid (up to 60%) has been observed, possibly due to protein–polyphenol interactions and the formation of bioactive peptides (Kautzmann et al. 2024). Technological strategies such as microencapsulation have also demonstrated improved protection and stabilization of phenolic compounds during gastrointestinal digestion (Clemente 2024). Furthermore, the use of biosorption on *S. cerevisiae* has been shown to enhance the postdigestion availability of total phenols and caffeine compared to crude extracts (Ribeiro et al. 2019). In contrast, studies using simple infusions or aqueous extracts report greater losses of phenolic compounds during digestion; however, these can lead to the formation of colonic metabolites with potential antioxidant activity, as demonstrated in fermentation models (Baeza et al. 2017; Correa et al. 2017).

The stability of flavonoids was more variable but generally lower than that of methylxanthines. Compounds such as rutin, quercetin, and kaempferol showed moderate retention, suggesting that their molecular structure may influence resistance to digestive conditions. Overall, the data indicate that although a significant fraction of yerba mate compounds is degraded or transformed during digestion, a relevant portion remains available for absorption or colonic metabolism, reinforcing the functional potential of the plant even after gastrointestinal processing. These findings also underscore the importance of evaluating not only the initial composition of samples but also their digestive stability to better estimate their actual bioactive impact in the body.

## 9 Final Considerations

*I. paraguariensis* stands out as a plant species of significant economic, cultural, and scientific importance, offering a wide spectrum of bioactive compounds with proven health‐promoting properties. Its broad range of applications from traditional beverages to cosmetic, pharmaceutical, and nutraceutical formulations reinforces its potential as a strategic ingredient for the innovation of functional and sustainable products.

This integrative review highlights that factors such as genotype, agronomic conditions, postharvest processing, and extraction methods significantly influence the plant's chemical profile. Moreover, studies employing simulated gastrointestinal digestion have contributed to understanding the bioaccessibility and stability of key compounds such as phenolic acids and methylxanthines, offering more realistic support for their biological efficacy.

Nonetheless, scientific gaps persist, particularly in the standardization of analytical methods and in systematic comparisons between cultivars, preparation forms, and extraction technologies. The incorporation of omics approaches and bioavailability assays using in vivo models may represent the next step toward consolidating evidence and validating functional claims.

The full valorization of *I. paraguariensis* thus requires integration between basic science, technological development, and specific regulations, along with public policies that encourage its innovative and sustainable use. This scenario strengthens the positioning of yerba mate not only as a South American cultural heritage but also as a high‐value global bioeconomic resource.

*I. paraguariensis* is a plant of considerable economic, cultural, and functional relevance, whose derivatives have gained attention not only in traditional contexts but also as promising ingredients for the development of foods, beverages, and formulations with bioactive properties. Its rich composition of phenolic acids, flavonoids, methylxanthines, and saponins supports well‐documented physiological effects, including antioxidant, anti‐inflammatory, lipid‐lowering, neurostimulant, and gastroprotective actions.

Consistent evidence shows that cultivar and cultivation systems strongly shape the phytochemical profile, while standardized processing and preparation methods are essential to ensure reproducibility and product quality. A key strength of this review is the integration of recent evidence on the in vitro gastrointestinal behavior of yerba mate, highlighting the stability and bioaccessibility of its bioactive compounds during simulated digestion. Incorporating such studies, still underexplored in the literature, offers a more realistic perspective on the functional delivery of compounds to the human body and represents a conceptual advance in the scientific valorization of the species.

Despite the advances, further studies are needed to integrate in vitro, in vivo, and metabolomic analyses to better elucidate the mechanisms of absorption, metabolism, and bioavailability of *I. paraguariensis* compounds. Additionally, there is room for the development of commercial cultivars with optimized phytochemical profiles and greater raw material standardization, especially for use in products with substantiated health claims.

## Author Contributions

**Sandra Maria Schenatto Palavicini**: methodology, conceptualization. **Bruna Maria Saorin Puton**: methodology. **Rosangela Assis Jacques**: validation, visualization. **Eunice Valduga**: validation, visualization. **Geciane Toniazzo Backes**: visualization, validation. **Natalia Paroul**: validation, investigation. **Clarice Steffens**: validation, formal analysis, supervision, writing—review and editing. **Rogério Luis Cansian**: supervision, writing—review and editing.

## Conflicts of Interest

The authors declare no conflicts of interest.

## Data Availability

Data will be made available upon request.
